# Interleukin-27 as a Diagnostic Biomarker for Patients with Sepsis: A Meta-Analysis

**DOI:** 10.1155/2021/5516940

**Published:** 2021-04-13

**Authors:** Ying Wang, Jingyi Zhao, Yinhui Yao, Dan Zhao, Shiquan Liu

**Affiliations:** ^1^Department of Pharmacy, The Affiliated Hospital of Chengde Medical College, Chengde 067000, China; ^2^Department of Functional Center, Chengde Medical College, Chengde 067000, China; ^3^Department of Intensive Care Unit, The Affiliated Hospital of Chengde Medical College, Chengde 067000, China; ^4^Department of Thoracic Surgery, The Affiliated Hospital of Chengde Medical College, Chengde 067000, China

## Abstract

**Background:**

The present study was aimed to investigate the value of blood interleukin-27 (IL-27) as a diagnostic biomarker of sepsis.

**Methods:**

We searched PubMed, EMBASE, the Cochrane Library, and the reference lists of relevant articles. All studies published up to October 21, 2020, which evaluated the accuracy of IL-27 levels for the diagnosis of sepsis were included. All the selected papers were assessed using the Quality Assessment of Diagnostic Accuracy Studies-2 (QUADAS-2). We used a bivariate random effects model to estimate sensitivity, specificity, diagnostic odds ratios (DOR), and a summary receiver operating characteristic curve (SROC). Deeks' funnel plot was used to illustrate the potential presence of publication bias.

**Results:**

This meta-analysis included seven articles. The pooled sensitivity, specificity, and DOR were 0.85 (95% CI, 0.72-0.93), 0.72 (95% CI, 0.42-0.90), and 15 (95% CI, 3-72), respectively. The area under the summary receiver operating characteristic curve was 0.88 (95% CI, 0.84-0.90). The pooled *I*^2^ statistic was 96.05 for the sensitivity and 96.65 for the specificity in the heterogeneity analysis. Deeks' funnel plot indicated no publication bias in this meta-analysis (*P* = 0.07).

**Conclusions:**

The present results showed that IL-27 is a reliable diagnostic biomarker of sepsis, but it should be investigated in combination with other clinical tests and results.

## 1. Introduction

Sepsis is a severe complication of severe infection, severe trauma, burns, shock, and surgery, and it can lead to septic shock and multiple organ dysfunction syndromes (MODS) [1, 2]. Currently, sepsis poses a significant public health effect in all parts of the world as it is associated with high morbidity and mortality. The morbidity rate in patients with sepsis is 29.5% in the hospital and 47% in the intensive care unit (ICU), and the mortality rate in patients with sepsis is 25.8% in the ICU and 35.3% in the hospital [3, 4]. When shock is present, the rates can increase to 40%~50% [5, 6]. Presently, the gold standard in diagnosing sepsis is microbiologic cultures, in spite of this is a delay between the clinical manifestations and the results from such cultures. As a result, patients do not receive timely antibacterial treatment, often leading to unfavorable outcomes [7–9]. Therefore, diagnostic biomarkers that can indicate a diagnosis of sepsis before the microbiological cultures are complete are needed.

Among the current diagnostic biomarkers used for sepsis, procalcitonin (PCT) is the most common. Several meta-analyses of the utility of PCT in diagnosing infection and sepsis have been performed. However, the usefulness of PCT is variable, and PCT is insufficient to distinguish between infected and uninfected critically ill patients [10–13]. Many studies have shown the efficacy of interleukin-27 (IL-27) for the differential diagnosis of sepsis and nonsepsis [14–18]. IL-27 is produced by antigen-presenting cells exposed to inflammatory stimuli and other conditions, and it is composed of EBI3 (an IL-12p40-related protein) and p28 (an IL-12p35-related polypeptide) joined with disulfide bonds. IL-27 induces the proliferation of naive CD4+ T cells and causes both proinflammatory and anti-inflammatory reactions [19, 20]. IL-27 plays a remarkable role in the evolution of various diseases through its involvement in antitumor immunity and anti-infection immunity. In animal experiments, neutralizing the biological function of IL-27 improves the survival time of mice with sepsis [21]. Hence, it is biologically feasible to use IL-27 as a diagnostic marker for sepsis.

To date, there has been no meta-analysis on the effectiveness of IL-27 as a biological marker for sepsis diagnosis. Therefore, this meta-analysis was the first to make a judgment about the diagnostic accuracy of IL-27 in sepsis and to evaluate the associated specificity and sensitivity.

## 2. Methods

### 2.1. Search Strategy

A systematic search was performed for relevant literature in three databases (the Cochrane Library, EMBASE, and PubMed). The correlational studies reported that IL-27 is a useful biomarker for the differential diagnosis of patients with sepsis and nonsepsis, and they were published prior to October 21, 2020. The search keywords were as follows: (interleukin-27 or IL-27) in combination with “sepsis” and “biomarker”. There were no language or publication-type restrictions. We checked the reference lists of correlational articles to confirm additional eligible studies not included in the databases. All the relevant articles were published.

### 2.2. Selection Criteria

In the meta-analysis, case-control studies using IL-27 for the differential diagnosis of patients with sepsis and nonsepsis were included. Then, the case-control studies provided details of data to construct 2 × 2 contingency tables. The exclusion criteria used were as follows: (1) meta-analyses, conference abstracts, correspondences, letters, case reports, editorials, animal experiments, and reviews; and (2) unavailable or insufficient data.

### 2.3. Data Extraction and Quality Assessment

Two authors, including the first author, independently extracted the following information from the eligible studies: the country where the research was conducted, the publication year, the numbers of cases and controls, biomarkers, and the data for the meta-analysis (true positive (TP), true negative (TN), false positive (FP), false negative (FN), sensitivity and specificity). We evaluated the quality of the included literature with the QUADAS-2 tool [22].

### 2.4. Statistical Analysis

To obtain the diagnostic accuracy of IL-27 for sepsis, we pooled the sensitivity, specificity, PLR, NLR, and DOR; we constructed a SROC curve and computed the area under the curve (AUC) with Meta-disc 1.4 (XI Cochrane Colloquium, Barcelona, Spain) and Stata 14.0 (StataCorp, College Station, TX) software. We assessed the statistical heterogeneity using *I*^2^ statistics and identified the impact of potential heterogeneity on the specificity and sensitivity through threshold analysis, and we evaluated the publication bias of the included studies by Deeks' regression test of funnel plot asymmetry [23]. *P* < 0.05 or *I*^2^ > 50% indicated statistically significant heterogeneity.

## 3. Results

### 3.1. Data Selection and Study Characteristics

**Figure 1:**
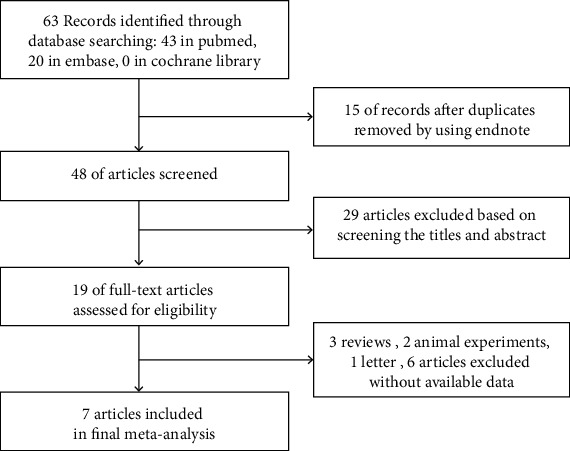
Seven studies collection flow chart.

The initial search retrieved 63 articles from the databases. After the duplicates were removed and the titles and abstracts were screened, we excluded 48 articles and provisionally included 19 articles. We made a full-text review of 7 articles and eliminated 12 articles [14–16, 24–27]. Finally, seven articles were used in our analysis (Figure 1). Seven studies included 589 sepsis patients and 535 controls, and they were performed in the USA, France, China, and Egypt. The included patient populations were neonates, children, and adults. The detailed characteristics of the seven studies included in our meta-analysis are listed in Table 1.

### 3.2. Quality of the Included Studies

**Figure 2:**
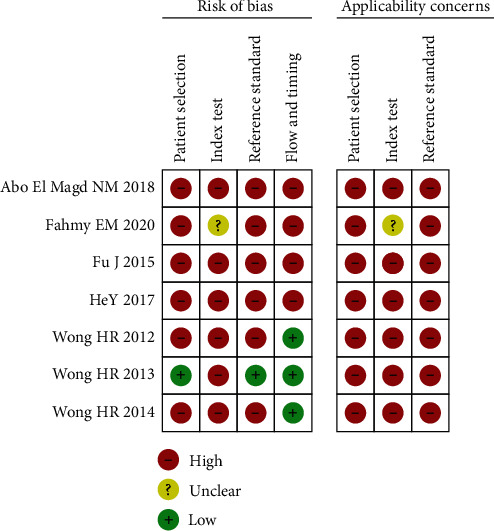
Risk of bias and applicability concerns of the included studies.

The QUADAS-2 tool was applied to evaluate the quality of the seven studies about IL-27 for differential diagnosis in patients with sepsis, and the detailed results are shown in Figure 2.

### 3.3. Diagnostic Accuracy

**Figure 3:**
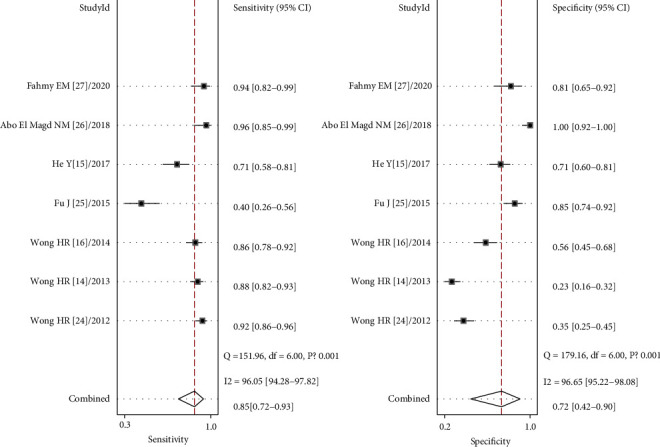
Forest plots of the sensitivity and specificity of interleukin-27 as a diagnostic biomarker for sepsis.

**Figure 4:**
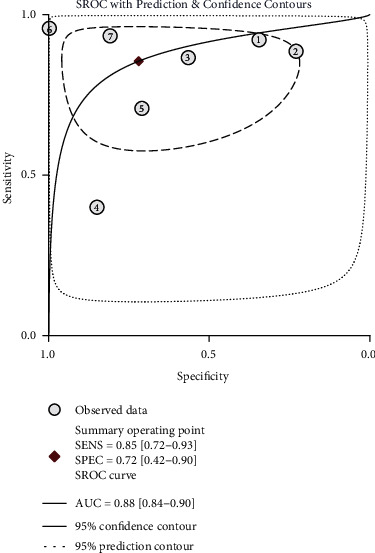
Symmetrical summary receiver operator curve (SROC) for interleukin-27 in all seven studies.

The following results were computed using the Stata 14.0 software: 0.85 (95% CI, 0.72-0.93) for sensitivity, 0.72 (95% CI, 0.42-0.90) for specificity (Figure 3), 15 (95% CI, 3-72) for DOR, 3.0 (95% CI, 1.2-7.7) for PLR, and 0.20 (95% CI, 0.09-0.46) for NLR. The pooled AUC was 0.88 (95% CI, 0.84-0.90), suggesting that IL-27 is a highly accurate diagnostic biomarker for sepsis (Figure 4).

### 3.4. Heterogeneity Analysis

We performed a heterogeneity analysis with the seven studies, and the *I*^2^ was 96.05 (95% CI, 94.28-97.82) for sensitivity and 96.65 (95% CI, 95.22-98.08) for specificity, indicating heterogeneity among the studies. The threshold analysis *P* value was 0.76, indicating no heterogeneity in our meta-analysis.

### 3.5. Publication Bias

**Figure 5:**
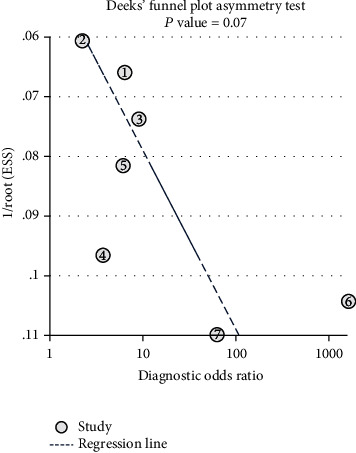
Deeks' funnel plot.

We evaluated publication bias through Deeks' funnel plot asymmetry test, which clearly demonstrated that no significant publication bias existed in this meta-analysis (*P* = 0.07) (Figure 5).

## 4. Discussion

Currently, PCT is a biomarker that is broadly used in clinical diagnosing for bacterial infection, but it cannot be used to distinguish sepsis from noninfectious causes of systemic inflammatory response syndrome (SIRS) in critical patients [11]. Genome-wide expression analysis has indicated that IL-27 combined with PCT is a better predictor of infection than either biomarker alone [24]. Moreover, Eckerle et al. showed that IL-27 is more effective than PCT in diagnosing bacterial infections in pediatric emergencies [28]. Studies have shown that IL-27 also links up with suppression of inflammation and that IL-27 is a therapeutic target for limiting neonatal susceptibility to sepsis and improving infection outcomes [29–32]. Wong et al. calculated the AUC and cut-off points, and they showed that IL-27 is comprehensively superior to PCT as a biomarker for diagnosing sepsis [24]. Therefore, our meta-analysis included seven articles to investigate the value of IL-27 in diagnosing patients with sepsis. The results of our study clearly demonstrated that IL-27 was an accurate diagnostic biomarker for sepsis with the potential for clinical applicability. In our meta-analysis, the sensitivity was 0.84, and the specificity was 0.71, indicating an adequate overall diagnostic accuracy. The SROC provides reliable summarized data of diagnostic studies, showing the intuitive trade-off between specificity and sensitivity. According to the results of SROC, the AUC was 0.88, which further heralded that the overall accuracy was good. The sensitivity, specificity, and AUC of IL-27 in our study showed similarities with PCT (0.77, 0.79, and 0.85, respectively) [12], IL-6 (0.73, 0.76, and 0.81, respectively) [33], and presepsin (0.77, 0.73, and 0.86, respectively) [13], which are valuable diagnostic markers for sepsis in published studies. Thus, IL-27 may develop into a biological marker in diagnosing sepsis in the future.

To evaluate the overall accuracy, the DOR integrates the sensitivity and specificity into a readily interpretable number [34]. The DOR represents the ratio of the probability of the positive results between the conditional group and unconditional group. The DOR varies from 0 to infinity and DOR values with a larger notice better diagnosis. When the value of DOR is less than 1.0, it indicates that the test is unable to distinguish the patients with or without the condition. In our meta-analysis, the calculated DOR was 15 (95% CI, 3-72), suggesting a high overall accuracy of IL-27 as a biological marker for differential diagnosis of sepsis.

We also performed a heterogeneity analysis to discover the existence of heterogeneity among the studies. The *I*^2^ value was 96.05 for sensitivity and 96.65 for specificity, indicating a high degree of heterogeneity among the studies. Several factors may have contributed to this high degree of heterogeneity. First, the age distributions of the subjects differed as adults, neonates, and children were included. Different populations have different pathophysiological characteristics. A previous study has shown that the average IL-27 levels in the plasma of children with sepsis are higher than those in adults because the upregulation of IL-27 expression in answer to infection is more robust in children [14, 16]. Thus, IL-27 may be a more useful diagnostic biomarker in patients with sepsis who were not yet an adult. Second, seven studies were performed in different regions, including the USA, France, China, and Egypt. The inclusion of different ethnicities can contribute to heterogeneity. Third, sepsis was defined differently in various studies [35]. The new definition of sepsis was published in 2016 [1]; four studies used the pre-2016 report, and two studies used the latest information. For clinically septic but culture-negative patients, the lack of a harmonized definition may lead to a high degree of heterogeneity. Fourth, the pathogenic bacteria differed among the patients. The post hoc analyses showed that the AUC for IL-27 in patients with secondary sepsis infection caused by a Gram-positive bacterium and Gram-negative bacterium was 0.639 and 0.768, respectively [14, 16]. Therefore, the bacterial etiology of sepsis should be considered as a source of heterogeneity.

**Figure 6:**
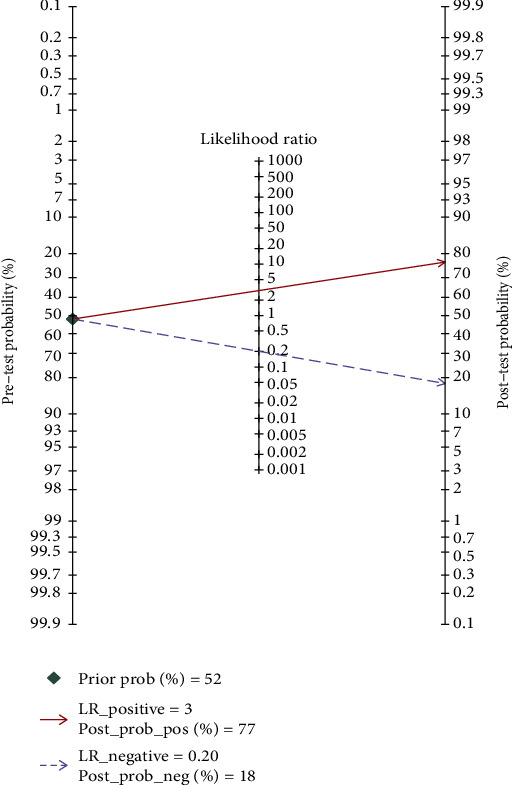
Fagan nomogram of interleukin-27 for the diagnosis of sepsis.

To increase the clinical applicability of the results of IL-27 in this meta-analysis, we used LRs to calculate the posttest probabilities according to Fagan's nomogram. Given a pretest probability of 52%, the pooled PLR of 3 increased the posttest likelihood (positive predictive value) to 77%. Thus, 77 of 100 patients with positive IL-27 results could be expected to have a confirmed diagnosis of sepsis. Moreover, the pooled NLR of 0.20 reduced the posttest probability to 18%. Thus, only 18 of 100 patients with negative IL-27 results may ultimately be diagnosed with sepsis (Figure 6). These results indicated that IL-27 is a useful diagnostic indicator for sepsis.

In this meta-analysis, several limitations attracted our attention and should be discussed. Primarily, the degree of heterogeneity should be considered. Due to the small sample size and the limited data available, we could not perform a meta-regression analysis. Therefore, we only analyzed factors underlying the existence of heterogeneity. Second, all databases used to identify the studies were in English. Therefore, some publications may have been overlooked, which may have been one of the causes of the bias in this meta-analysis. To overcome the above problems, the authors repeated the search to include more studies but did not find additional relevant articles or available data.

## 5. Conclusions

In conclusion, IL-27 can be used as a candidate biomarker for the diagnosis of patients with sepsis. Although IL-27 has the potential for clinical applicability for identifying sepsis in critical patients, the test results of IL-27 should be explained in combination with clinical and other test factors. Further studies with a large sample size are needed to be designed to reduce the heterogeneity in the analysis of the utility of IL-27 for discrimination between sepsis and nonsepsis.

## Figures and Tables

**Table 1:** Characteristics of the included studies.

Author	Year	Country	Cases/controls	Cutoff, ng/mL	TP	FN	FP	TN	Sensitivity	Specificity	Population
Wong HR [24]	2012	USA	130/101	2	120	10	66	35	92%	35%	Children
Wong HR [14]	2013	France	145/125	1	128	17	96	29	88%	23%	Adults
Wong HR [16]	2014	USA	109/78	1	94	15	34	44	86%	56%	Adults
Fu J [25]	2015	China	45/66	3.6	18	27	10	56	40%	85%	Adults
He Y [15]	2017	China	68/83	1	48	20	24	59	70.59%	71.08%	Neonates
Abo El Magd NM [26]	2018	Egypt	45/45	485.56	43	2	0	45	95.56%	100%	Neonates
Fahmy EM [27]	2020	Egypt	47/37	—	44	3	7	30	93.6%	81.1%	Neonates

## Data Availability

The data of Table 1 used to support the findings of our study are included within the article (see References).
